# Optical characterisation of silicon nanocrystals embedded in SiO_2_/Si_3_N_4 _hybrid matrix for third generation photovoltaics

**DOI:** 10.1186/1556-276X-6-612

**Published:** 2011-12-03

**Authors:** Dawei Di, Heli Xu, Ivan Perez-Wurfl, Martin A Green, Gavin Conibeer

**Affiliations:** 1 ARC Photovoltaics Centre of Excellence, University of New South Wales, Sydney, NSW 2052, Australia

**Keywords:** silicon nanocrystals, third generation photovoltaics, absorption coefficient, photoluminescence, band gap extraction

## Abstract

Silicon nanocrystals with an average size of approximately 4 nm dispersed in SiO_2_/Si_3_N_4 _hybrid matrix have been synthesised by magnetron sputtering followed by a high-temperature anneal. To gain understanding of the photon absorption and emission mechanisms of this material, several samples are characterised optically via spectroscopy and photoluminescence measurements. The values of optical band gap are extracted from interference-minimised absorption and luminescence spectra. Measurement results suggest that these nanocrystals exhibit transitions of both direct and indirect types. Possible mechanisms of absorption and emission as well as an estimation of exciton binding energy are also discussed.

## Background

Self-assembled silicon nanocrystals [Si NCs] embedded in a dielectric matrix are believed to be a promising material for applications in optoelectronics [1-3] and photovoltaic solar cells [4-10]. One major advantage of Si nanocrystals over bulk Si is the freedom to engineer the material's effective band gap by varying the size of the Si NCs or by modifying the properties of the matrix material. A simple method of fabricating 'SiO/SiO_2 _superlattice' or 'Si NCs in SiO_2 _matrix' was described by Zacharias et al. [11]. The optical absorption properties of this kind of superlattices were investigated by a number of groups [12-14]. Photovoltaic diodes fabricated using similar approaches have been demonstrated by some of the present authors [5,6]. Their limitations include high device resistivity and the lower-than-expected output voltages.

To overcome these problems, an improved nanostructure, 'Si NCs in SiO_2_/Si_3_N_4 _hybrid matrix', has been recently proposed by us for the application of 'Si quantum dot photovoltaics' [7]. Experimental investigations have shown that the material possesses better nanocrystal growth and carrier transport properties [8]. However, few studies have been conducted to comprehensively examine the new material's optical characteristics, which are essential in the understanding of device operation. In this paper, we report some initial experimental observations on the optical properties of Si NCs embedded in a SiO_2_/Si_3_N_4 _hybrid matrix.

### Experimental details

**Figure 1:**
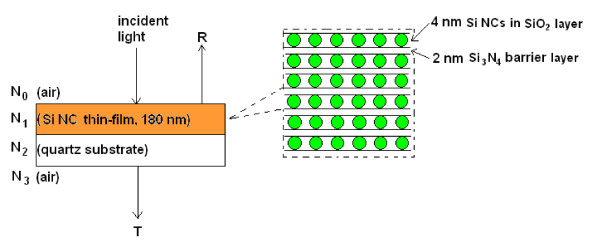
**Schematic diagram of the sample structure**. *N*_k _denotes the complex refractive index of the corresponding medium.

Alternating layers of a 2-nm Si_3_N_4 _followed by a 4-nm doped silicon-rich oxide [SRO] were deposited on quartz substrates by magnetron sputtering of Si_3_N_4_, Si, SiO_2 _and dopant targets using a computer-controlled AJA ATC-2200 sputtering system (AJA International, Inc. Scituate, MA, USA). The total number of bilayers is 30, making the total thickness of the deposited thin films to be approximately 180 nm. The volume ratio between the co-sputtered Si and SiO_2 _was 1.2:1 as determined by a built-in deposition rate monitor. Dopant species such as boron [B] or phosphorus pentoxide [P_2_O_5_] were incorporated into the SRO layers during the sputtering process. Prior to sputtering, the chamber of the sputtering system was evacuated to a pressure of approximately 5 × 10^-7 ^Torr. Subsequently, the chamber was filled with Ar gas to a working pressure of 1.5 × 10^-3 ^Torr. The Ar flow was maintained at 15 sccm during the entire deposition process. After the deposition process, the samples were annealed in a N_2_-purged tube furnace at 1,100°C to facilitate Si NC growth. The intended sample structure is illustrated in Figure 1.

**Figure 2:**
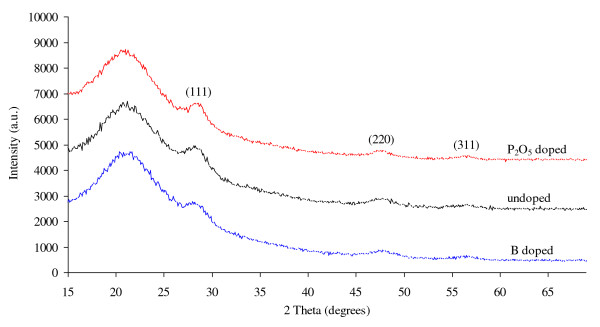
**XRD patterns of samples investigated in this work**.

The crystalline properties of the samples were studied by glancing angle incidence X-ray diffraction [XRD] (Phillips X'pert Pro, PANalytical B.V., Almelo, The Netherlands) using Cu Kα radiation (*λ *= 0.154 nm), operating at a voltage of 45 kV and a current of 40 mA (The results are shown in Figure 2). The primary optics was defined by using a 1/16° divergent slit in front of a parabolic mirror. The secondary optics consists of a parallel plate collimator of 0.27° acceptance and a Soller slit of 0.04 rad aperture. The measured X-ray results correspond to an average sample area of about 20 × 20 mm^2^. The glancing angle between the incident X-ray beam and the sample surface was set to be at 0.255° i.e., close to the critical angle. The photoluminescence [PL] of the samples was studied at room temperature using a 540-nm laser as the excitation source. A dual-beam UV/visible/IR spectrometer (Varian Cary 5G, Varian Inc., Palo Alto, CA, USA) was used to measure optical transmission (*T*) and reflection (*R*) spectra.

## Analysis and discussion

A set of equations which is able to calculate the complex refractive indices (*N *= *n *+ *ik*) from the *R *and *T *data was derived by Hishikawa et al. for the analysis of a-Si thin films [15]. This equation set (Equations 1 to 10), listed as follows, is able to minimise the influence of thin-film interference effects [15] and thus is also applicable in the analysis of Si NC materials.

(1) $$T=\frac{T_{23}T_{02}}{1-R_{20}R_{23}}$$

(2) $$R=\frac{T_{20}^{2}R_{23}}{1-R_{20}R_{23}}+R_{02}$$

(3) $$\begin{array}{ccc} {\left(\frac{T}{1-R}\right)}^{-1} & =\frac{\left(1-R_{02}\right)\left(1-R_{20}R_{23}\right)-T_{{}_{20}}^{2}R_{23}}{T_{23}T_{02}} & \\ & =\frac{1-R_{02}}{T_{23}T_{02}}-\frac{R_{23}}{T_{23}}\left(R_{20}\frac{1-R_{02}}{T_{02}}+T_{20}\right) \end{array}$$

(4) $$T_{02}=T_{20}=\frac{n_{2}}{n_{0}}{\left|\frac{e_{1}t_{01}t_{12}}{1-e_{1}^{2}r_{10}r_{12}}\right|}^{2}$$

(5) $$R_{02}={\left|r_{01}+\frac{e_{1}^{2}t_{01}t_{10}r_{12}}{1-e_{1}^{2}r_{10}r_{12}}\right|}^{2}$$

(6) $$R_{20}={\left|r_{21}+\frac{e_{1}^{2}t_{21}t_{12}r_{10}}{1-e_{1}^{2}r_{12}r_{10}}\right|}^{2}$$

(7) $$T_{23}={\left|t_{23}\right|}^{2}\frac{n_{3}}{n_{2}},\;R_{23}={\left|r_{23}\right|}^{2},$$

(8) $$e_{1}=\exp\left(\frac{2i\pi N_{1}d}{\lambda }\right),$$

(9) $$t_{kl}=\frac{2N_{k}}{N_{k}+N_{l}},\;r_{kl}=\frac{N_{k}-N_{l}}{N_{k}+N_{l}},$$

(10) $$N_{k}=n_{k}+ik_{k}:\;\text{complex refractive index of medium }k.$$

**Figure 3:**
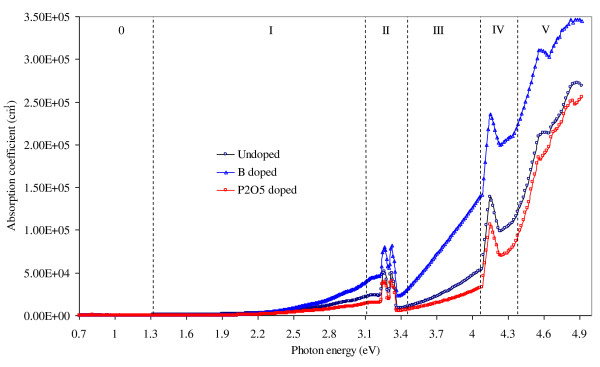
**Absorption coefficients as functions of incident photon energy for samples with different doping**.

**Figure 4:**
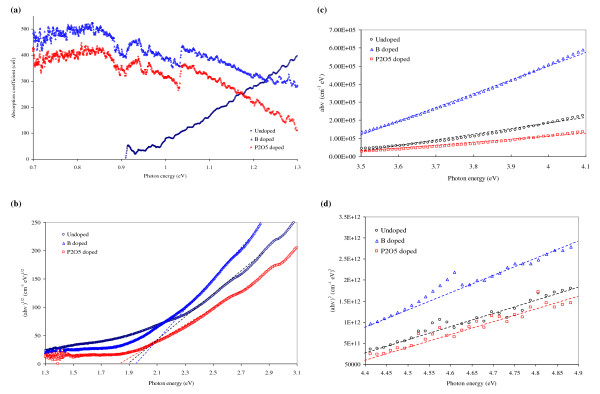
**Absorption coefficient curves and Tauc plots**. **(a) **Absorption coefficient curves in region 0 of Figure 3; **(b) **Tauc plot of region I with *γ *= 1/2. The dashed lines are fittings to the quasi-linear parts of the curves; **(c) **Tauc plot of region III with *γ *= 1; **(d) **Tauc plot of region V with *γ *= 2.

Following the above calculation, the absorption coefficient of the material at each photon wavelength can then be obtained by *α *(*λ*) = 4π*k/λ*. We have also incorporated film thickness calculations in our analysis. This approach was originally suggested by Hishikawa et al. [15] and was realised in our calculation programme. The fitting results indicate that the actual thickness of the films falls in the range of 177 to approximately 186 nm, which is very close to its nominal value (180 nm). The absorption coefficients of undoped, B- and P_2_O_5_-doped Si NCs in SiO_2_/Si_3_N_4 _hybrid matrix materials determined using the above method for photon energies ranging from 0.7 eV to 5 eV are shown in Figure 3. For convenience, we divide the absorption curves into six different regions (regions 0 to V). Across all regions, the B-doped sample shows generally larger absorption coefficients than the undoped and the P_2_O_5_-doped samples. This is most likely due to the reason that the B-doped samples contain, on average, smaller Si NCs (average NC sizes measured by XRD (Figure 2): B-doped = 3.5 nm, P_2_O_5_-doped = 5 nm, undoped = 4.3 nm), which results in a higher cross-sectional density of NCs than samples with larger grains. A close-up view of region 0 is shown in Figure 4a. It is interesting to note that the intentionally doped Si NC films are more optically absorbing than the undoped material in this photon energy range (0.7 to approximately 1.3 eV). These absorption tails show characteristics of free-carrier absorption related to heavy doping effects [16] and provide evidence of successful dopant incorporation in Si NCs.

Region I is a region in which the absorption curves generally exhibit square dependence. By applying the Tauc analysis (in its generalised form: (*αhν*)*^γ ^*versus *hν*) on region I and take *γ *= 1/2, the resultant graph is shown in Figure 4b. The intercepts of the quasi-linear sections on the energy axis represent the band gaps extracted from the optical absorption measurements. The band gaps are of indirect nature, as *γ *= 1/2 is used to obtain the linearised spectra [17,18]. The estimated first indirect gaps are 1.90 eV, 1.95 eV and 1.84 eV for undoped, B-doped and P_2_O_5_-doped samples, respectively. This transition, although about 0.78 eV higher in energy due to quantum confinement, can be related to the first indirect transition (Г_25_' - X_1_) in Si.

The absorption curves in region III are mostly linear. Therefore, Tauc plots with *γ *= 1 are best suited for the analysis (Figure 4c). The linear extrapolations cross the energy axis at around 3.4 eV. Since *γ *= 1, and thus 1/2 <*γ *< 2, the photon absorption that occurs in this region is a 'quasi-direct' transition. We assign this to the joint contribution of the indirect (Г_25_' - L_1_) and the direct (Г_25_' - Г_15_) transitions.

In region V, the lower density of data acquisition and the instrument's measurement limit lead to some uncertainty in the analysis. However, the absorption curves in this region generally follow a square-root dependence. Thus by taking *γ *= 2 in the generalised Tauc analysis (Figure 4d), we obtain *x*-intercepts in the photon energy region of 4.1 to 4.3 eV. These absorption bands resemble direct transitions (*γ *= 2) [18]. The average value of the energy gaps (4.2 eV) is comparable with the direct transition (Г_25_' - Г_2_') in unconfined Si. However, it should be noted that the Tauc analysis may not be strictly applicable because it assumes parabolic energy bands. This is not necessarily the case for NCs and is the reason for the mixed direct/indirect nature of the analysis presented here.

The absorption peaks in regions II, IV and V have not been clearly understood. Since they appear at certain energies regardless of the kind of dopant introduced, they are likely due to measurement errors or defect states. The measurement error of our spectrometer is within 2%, as specified by the manufacturer. The main sources of experimental error include different sample placements in reflection and transmission modes as well as the change of detector/source during measurement. However, the influence of these factors on the accuracy of the optical band gap estimation is very small because of the following reasons: (1) the analysis method we presented in this paper calculates absorption coefficient versus wavelength data on a point-by-point basis, which means each data point is analysed separately so that errors or noises present in particular points do not affect the analysis of their neighbouring points; and (2) to further eliminate the effects of instrumental errors and noises, we examine only the non-abrupt and relatively smooth regions (e.g., I, II and V) of the absorption curves.

**Figure 5:**
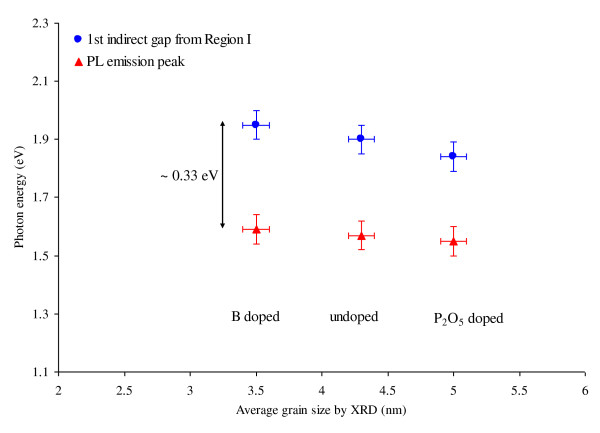
**Tauc band gaps and positions of PL peaks**. The first indirect gaps extracted from the absorption spectra and the positions of PL peaks as functions of average nanocrystal grain size measured by XRD.

What is also of interest is to compare the first indirect band gaps extracted from region I with the peak energies of PL emission spectra (Figure 5). It can be seen that as the size of the Si NC deceases, the first optical band gap and the PL peak gradually shift toward higher energies. This behaviour is a manifestation of quantum confinement and is consistent with our previous investigations [6,7]. It is important to note that the average value of the first indirect gap obtained from the optical absorption is 1.90 eV, while the average PL peak position of the same samples is 1.57 eV. The discrepancy of about 0.33 eV between the two values is possibly attributed to defect bands or is a measure of exciton binding energy. The latter is more likely to be the case due to the very gradual blue shift with decreasing NC size.

## Conclusions

In conclusion, we have synthesised approximately 4-nm Si NCs of different dopant inclusions (B, P_2_O_5 _and undoped) dispersed in SiO_2_/Si_3_N_4 _hybrid matrix by magnetron sputtering followed by a high temperature anneal. Analyses of the interference-free optical absorption and photoluminescence spectra reveal that the direct/indirect character of the Si NCs is mixed. Based on the absorption spectra, the materials appear to have an indirect band gap at about 1.90 eV, a quasi-direct band gap at 3.4 eV and a direct gap at around 4.2 eV. The PL emission of these NCs occurs at around 1.57 eV, suggesting sub-band gap radiative transitions. A possible estimate of the exciton binding energy is around 0.33 eV. Future works could include the following: (1) improvement of material properties by defect passivation techniques, (2) fabrication of working devices based on these materials and (3) investigation on photocarrier lifetime and charge distribution in the devices.

## Abbreviations

PL: photoluminescence; Si NC: silicon nanocrystal; SRO: silicon-rich oxide; XRD: X-ray diffraction.

## Competing interests

The authors declare that they have no competing interests.

## Authors' contributions

DD fabricated the Si NC samples, carried out measurements, analyzed the data and drafted the manuscript. HX conducted the optical measurements of the samples. IPW participated in the experimental design and calculations. GC and MAG supervised the work and helped improve the manuscript. All authors read and approved the final manuscript.
